# Structural, Hirshfeld surface and three-dimensional inter­action energy studies of 2-(6-iodo-4-oxo-3,4-di­hydro­quinazolin-3-yl)ethane­sulfonyl fluoride

**DOI:** 10.1107/S205698902201221X

**Published:** 2023-01-06

**Authors:** D. P. Ganesha, N. R. Sreenatha, S. R. Shankara, B. N. Lakshminarayana

**Affiliations:** a Department of Engineering Physics, Adichunchanagiri Institute of Technology, Chikkamagaluru - 577102, Karnataka, India; b Department of Physics, Government Engineering College, B M Road, Dairy Circle, Hassan - 573 201, Karnataka, India; c Department of Engineering Physics, BGS Institute of Technology, Adichunchanagiri University, B G Nagara, Karnataka, India; University of Durham, United Kingdom

**Keywords:** Single-crystal structure, C—H⋯N and C—H⋯O hydrogen bonds, I⋯O halogen bond, Hirshfeld surface, inter­molecular energies

## Abstract

In the crystal, mol­ecules of the title compound, C_10_H_8_FIN_2_O_3_S, are connected through C—H⋯N and C—H⋯O hydrogen bonds, I⋯O halogen bonds, π–π stacking inter­actions between the benzene and pyrimidine rings, and edge-to-edge electrostatic inter­actions, as shown by the analysis of the Hirshfeld surface and two-dimensional fingerprint plots, as well as inter­molecular inter­action energies calculated using the electron-density model at the HF/3–21 G level of theory.

## Abstract 2

In the crystal, mol­ecules of the title compound are connected through C—H⋯N and C—H⋯O hydrogen bonds, I⋯O halogen bonds, π–π stacking inter­actions between the benzene and pyrimidine rings, and edge-to-edge electrostatic inter­actions, as shown by the analysis of the Hirshfeld surface and two-dimensional fingerprint plots, as well as inter­molecular inter­action energies.

## 1. Chemical context

Quinazoline is an aromatic heterocycle consisting of a benzene ring fused with a pyrimidine ring. Its derivatives are well known for their biological activities such as anti-analgesic, anti-inflammatory, anti-hypertensive, sedative, hypnotic, anti-histaminic, anti-tumor, anti-microbial, anti-convulsant, anti-bacterial, anti-fungal, enzyme inhibition, and anti-HIV activities (Kumar *et al.*, 1981 ▸; Baker *et al.*, 1952 ▸; Rewcastle *et al.*, 1995 ▸; Hitkari *et al.*, 1995 ▸; Bertelli *et al.*, 2000 ▸; Yang *et al.*, 2009 ▸; Cao *et al.*, 2009 ▸; De Clercq, 2001 ▸). Compounds bearing the quinazoline moiety also are potent cytotoxic agents (Ibrahim *et al.*, 1988 ▸; Riou *et al.*, 1991 ▸; Braña *et al.*, 1994 ▸; Helissey *et al.*, 1994 ▸), show anti-oxidant (Al-Amiery *et al.*, 2014 ▸) and insecticidal (Yang *et al.*, 2021 ▸) activities. In view of their therapeutic importance, we report herein the crystal structure, Hirshfeld surface and three-dimensional inter­action energy studies of 2-(6-iodo-4-oxo-3,4-di­hydro­quinazolin-3-yl)ethane­sulfonyl fluoride, (I). 
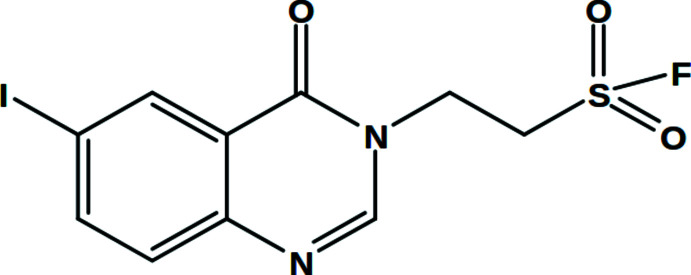


## 2. Structural commentary

**Figure 1:**
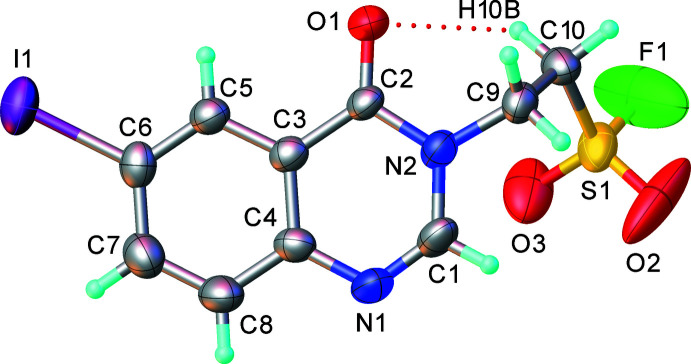
Mol­ecular structure of (I). The atomic displacement ellipsoids are drawn at the 50% probability level.

The mol­ecular structure of (I) (Fig. 1 ▸) shows an out-of-plane conformation of the (CH_2_)_2_SO_2_F side chain, the C9/C10/S1 fragment forming a dihedral angle of 76.1 (5)° with the quinazoline (N1/N2/C1–C8) system mean plane, whereas the I1 and O1 substituents do not deviate appreciably from the latter plane. The mol­ecule is stabilized by a weak intra­molecular C10—H10*B*⋯O1 hydrogen bond, forming an *S*(6) ring motif. The S1 atom has a slightly distorted tetra­hedral geometry. In the heterocycle, the N1=C1 bond [1.271 (8) Å] is essentially double, while those at the three-coordinate N2 atom are nominally single [C1—N2 = 1.359 (7), N2—C2 = 1.384 (6) Å].

The bond lengths and angles are in agreement with those in related structures (El-Hiti *et al.*, 2014 ▸; Al-Salahi *et al.*, 2012 ▸; Utayeva *et al.*, 2013 ▸; Priya *et al.*, 2011 ▸; Lakshminarayana *et al.*, 2009 ▸, 2022 ▸; Sreenatha *et al.*, 2018*a* ▸,*b* ▸, 2020 ▸, 2022 ▸).

## 3. Supra­molecular features

**Figure 2:**
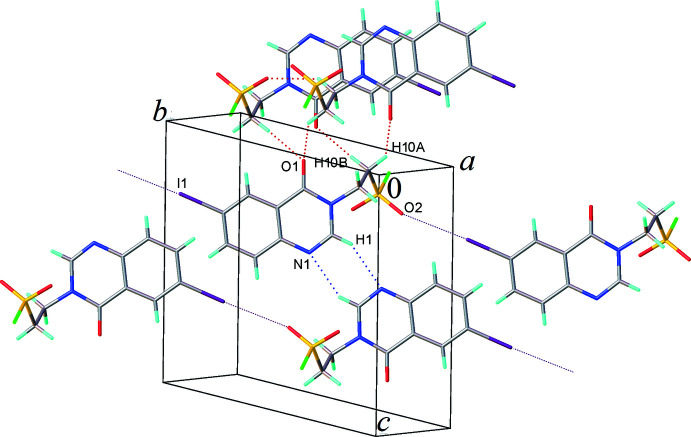
Inter­molecular hydrogen (see Table 1 ▸) and halogen bonds in the structure of (I).

**Figure 3:**
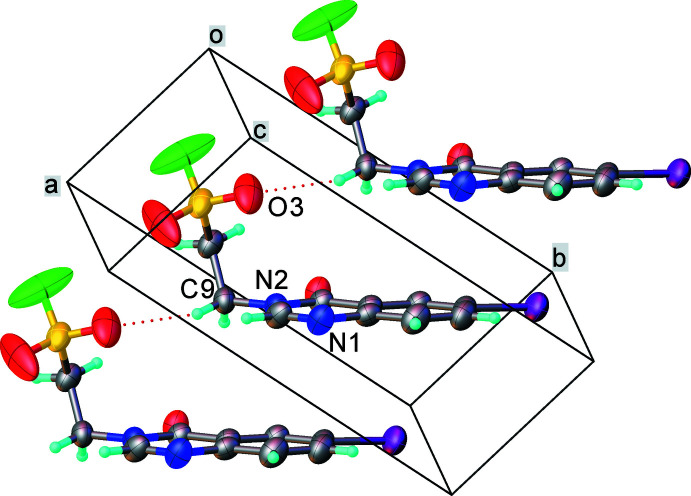
π–π stacking in the structure of (I).

In the crystal, each mol­ecule donates three and accepts three inter­molecular hydrogen bonds, *viz*. C1—H1⋯N1, C10—H10*B*⋯O1, C10—H10*A*⋯O1 (Table 1 ▸) and their inversion equivalents. Thus, each mol­ecule participates in three centrosymmetric dimers with 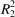 (6), 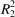 (12) and 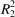 (12) ring motifs, respectively (Fig. 2 ▸). Mol­ecules related by the *a* translation, form a continuous stack *via* π–π inter­actions between the benzene and the pyrimidine rings (which are parallel within 1.5°), with a mean inter­planar separation of 3.503 (4) Å (Fig. 3 ▸). The I1⋯O2(*x* − 1, *y* + 1, *z*) contact of 3.152 (6) Å is considerably shorter than the sum of the van der Waals radii of 3.61 Å (Batsanov, 1995 ▸; Rowland & Taylor, 1996 ▸) and can be described as a halogen bond (Metrangolo & Resnati, 2001 ▸), the nearly linear angle C6—I1⋯O2 = 175.9 (3)° being typical of such bonds.

## 4. Database survey

A survey of the Cambridge Structural Database (CSD version 5.41, update of October 2022; Groom *et al.*, 2016 ▸) revealed only one structure, namely (*Z*)-ethyl-2-cyano-2-(3*H*-quinazoline-4-yl­idene)acetate (ACEZUE; Tulyasheva *et al.*, 2005 ▸), which shares such features of (I) as one two-coordinate (N1) and one three-coordinate (N2) nitro­gen atom of the quinazoline ring system, as well as an exocyclic double bond at C2, although in this case the H atom at N2 is not substituted. Of the other comparable quinazoline derivatives, in 3-amino-6-bromo-1-methyl-2, 4-(1*H*,3*H*)-quinazolinedione (ABMQZD; Ardebili & While, 1978 ▸) both N atoms are three-coordinate, while in *N*-(5-methyl-1,2-oxazol-3-yl)-4-[(quinazolin-4-yl) level of theoryamino]­benzene-1-sulfonamide and *N*-(3,4-dimethyl-1,2-oxazol-5-yl)-4-[(quinazolin-4-yl)amino]­benzene-1-sulfonamide (GEY­YOB, GEYYUH; Sunil Kumar *et al.*, 2018 ▸) both are two-coordinate.

## 5. Hirshfeld surfaces and 2D fingerprint calculations

**Figure 4:**
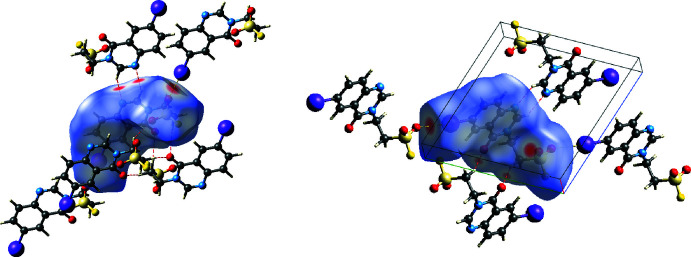
Different aspects of the three-dimensional Hirshfeld surface of (I) mapped over *d*_norm_. Red spots indicate shortened contacts, revealing inter­molecular hydrogen and halogen bonds

**Figure 5:**
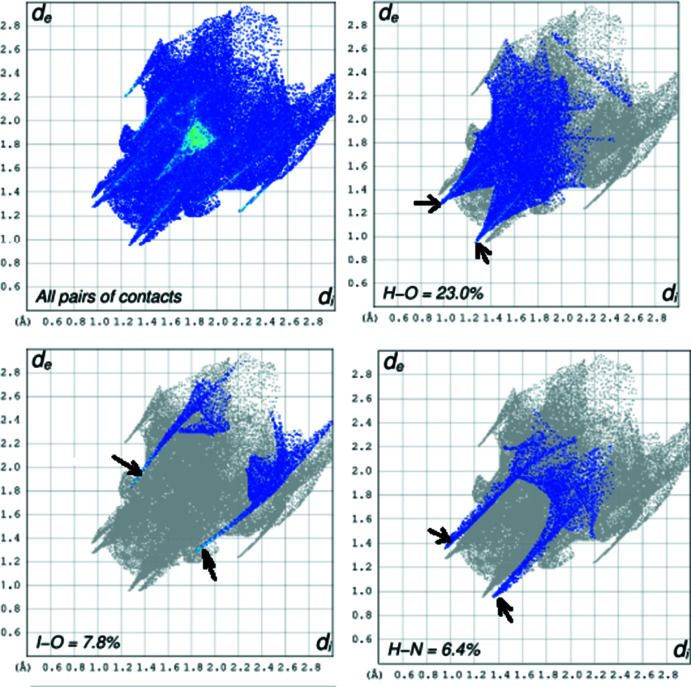
Selected two-dimensional fingerprint plots of structure (I); *d*_i_ and *d*_e_ are the distances from the Hirshfeld surface to the nearest inter­nal and external atoms. Arrows indicate the ‘spikes’ characteristic of hydrogen and halogen bonds

The Hirshfeld surfaces and two-dimensional fingerprint plots were calculated using *CrystalExplorer17.5* (Spackman *et al.*, 2009 ▸) to analyse the inter­molecular inter­actions. The three-dimensional Hirshfeld surface mapped over the normalized contact distance (*d*_norm_) is shown in Fig. 4 ▸. The eight bright-red spots, indicating shortened contacts, correspond to the three pairs of inter­molecular hydrogen bonds and one pair of halogen bonds discussed in Section 3. The two-dimensional fingerprint plots show the H⋯O contacts to be the most common (23.0%), followed by H⋯H (13.5%), H⋯C (11.5%), H⋯I (9.9%), I⋯O (7.8%), H⋯F (6.7%), H⋯N (6.4%), I⋯F (4.0%), I⋯C (3.2%), O⋯O (2.2%) and C⋯N (1.9%) (including the reverse ones for all heteronuclear contacts). The characteristic spikes in the plots of the H⋯O and H⋯N contacts indicate inter­molecular hydrogen bonds, those in the I⋯O plot indicate halogen bonds (Fig. 5 ▸).

## 6. Three-dimensional framework analysis of inter­action energies

**Figure 6:**
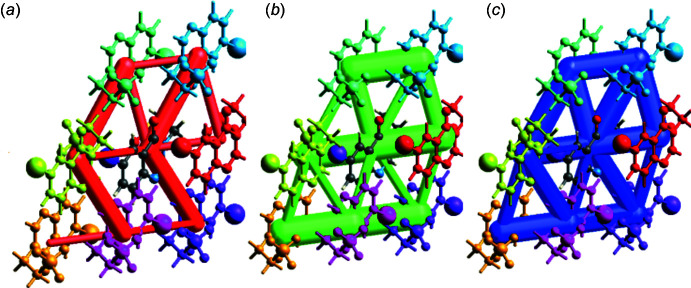
Inter­molecular energy frameworks of (*a*) *E*_ele_, (*b*) *E*_dis_ and (*c*) *E*_tot_ in the structure of (I), viewed down the *b* axis.

**Figure 7:**
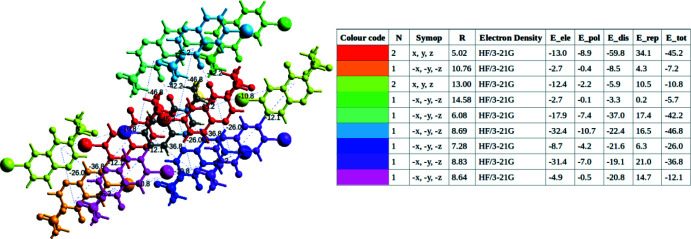
Inter­molecular energies (in kJ mol^−1^) and their components in the structure of (I). *N* is the number of mol­ecules in a group, *Symop* is the symmetry operator, *R* is the distance between mol­ecular centroids in Å.

Qu­anti­fication of inter­molecular inter­actions energies is important for mol­ecular recognition, protein modelling and drug design (Volkov & Coppens, 2004 ▸). We computed these energies for (I) with the HF/3-21G(d,p) electron-density model (Grimme, 2006 ▸), using *CrystalExplorer17.5* software. Eleven mol­ecules surrounding the original one with shortest inter­molecular atom–atom distances of 3.8 Å or less were included in the calculations. The total inter­action energy (*E*_tot_) between each pair of mol­ecules comprises coloumbic (*E*_ele_), dispersion (*E*_dis_), polarization (*E*_pol_) and exchange-repulsion inter­action energies (*E*_rep_) (Turner *et al.*, 2015 ▸, 2017 ▸). The *E*_ele_, *E*_dis_ and *E*_tot_ inter­molecular energy frameworks for (I) are shown graphically in Fig. 6 ▸ and numerically in Fig. 7 ▸. The mol­ecular stacks (Fig. 3 ▸, top line in the Fig. 7 ▸ table) are held together mostly by dispersion (van der Waals) inter­actions, supported by the shortest C—H⋯O hydrogen bonds, while edge-to-edge inter­molecular contacts (lines 5 to 8) have larger contributions of electrostatic inter­actions. The inter­action between halogen-bonded mol­ecules (line 3) is smaller than the above in absolute terms (10.8 kJ mol^−1^), but is remarkable given that only one pair of atoms is actually in contact.

## 7. Synthesis and crystallization

To an ice-cooled stirred suspension of NaH (60% suspension in mineral oil; 125 mg, 2.0 mmol, 2.0 equiv) and 6-iodo­quinazolin-4(3*H*)-one (1.0 mmol, 1.0 equiv) in DMF (2 mL), a solution of 2-bromo­ethane­sulfonyl fluoride (350 mg, 1.0 mmol, 1.0 equiv) in DMF (1 mL) was added, under an N_2_ atmosphere. The reaction was heated at 353 K for 4 h under an N_2_ atmosphere (monitored by TLC). After the complete conversion of the reactants as confirmed from TLC analysis, the reaction mixture was quenched with saturated NH_4_Cl solution (25 mL), extracted with EtOAc (25 mL) and the collected organic layer was further washed with water (25 mL) and brine (25 mL), then dried over anhydrous Na_2_SO_4_ and concentrated under vacuum. Compound (I) was isolated by silica gel chromatography (using chloro­form and methanol as mobile phase) and recrystallized from DMF.

## 8. Refinement

Crystal data, data collection and structure refinement details are summarized in Table 2 ▸. Hydrogen atoms were placed in idealized positions and refined using a riding model with C—H 0.93 Å for *sp* ^2^ and 0.97 Å for *sp* ^3^ C atoms, with *U*_iso_(H) = 1.2*U*_eq_(C) for both.

## Supplementary Material

Crystal structure: contains datablock(s) I. DOI: 10.1107/S205698902201221X/zv2021sup1.cif

Structure factors: contains datablock(s) I. DOI: 10.1107/S205698902201221X/zv2021Isup3.hkl

Supporting information file. DOI: 10.1107/S205698902201221X/zv2021Isup3.cml

CCDC reference: 1987361

Additional supporting information:  crystallographic information; 3D view; checkCIF report

## Figures and Tables

**Table 1:** Hydrogen-bond geometry (Å, °)

*D*—H⋯*A*	*D*—H	H⋯*A*	*D*⋯*A*	*D*—H⋯*A*
C1—H1⋯N1^i^	0.93	2.46	3.284 (8)	148
C10—H10*B*⋯O1^ii^	0.97	2.56	3.493 (7)	160
C10—H10*A*⋯O1^iii^	0.97	2.45	3.151 (7)	129
C9—H9*A*⋯O3^iv^	0.97	2.33	3.150 (8)	141
C10—H10*B*⋯O1	0.97	2.59	3.121 (7)	115

Symmetry codes: (i) 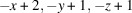 ; (ii) 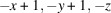 ; (iii) 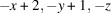 ; (iv) 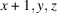 .

**Table 2:** Experimental details

Crystal data	
Chemical formula	C_10_H_8_FIN_2_O_3_S
*M*_r_	382.14
Crystal system, space group	Triclinic, *P* 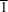
Temperature (K)	293
*a*, *b*, *c* (Å)	5.0230 (5), 11.3241 (11), 11.5509 (11)
α, β, γ (°)	103.081 (2), 96.742 (1), 97.860 (1)
*V* (Å^3^)	626.43 (11)
*Z*	2
Radiation type	Mo *K*α
μ (mm^−1^)	2.74
Crystal size (mm)	0.34 × 0.30 × 0.27

Data collection	
Diffractometer	Bruker APEXII
Absorption correction	–
No. of measured, independent and observed [*I* > 2σ(*I*)] reflections	4073, 3207, 2315
*R*_int_	0.035
(sin θ/λ)_max_ (Å^−1^)	0.673

Refinement	
*R*[*F* ^2^ > 2σ(*F* ^2^)], *wR*(*F* ^2^), *S*	0.055, 0.166, 1.11
No. of reflections	3207
No. of parameters	164
H-atom treatment	H-atom parameters constrained
Δρ_max_, Δρ_min_ (e Å^−3^)	0.88, −1.39

Computer programs: *APEX2* and *SAINT* (Bruker, 2009 ▸), *SHELXT* (Sheldrick, 2015*a* ▸), *SHELXL2018/3* (Sheldrick, 2015*b* ▸) and *PLATON* (Spek, 2020 ▸).

## supplementary crystallographic information

### Crystal data

**Table:**

C_10_H_8_FIN_2_O_3_S	*Z* = 2
*M**_r_* = 382.14	*F*(000) = 368
Triclinic, *P*1	*D*_x_ = 2.026 Mg m^−^^3^
*a* = 5.0230 (5) Å	Mo *K*α radiation, λ = 0.71073 Å
*b* = 11.3241 (11) Å	Cell parameters from 3207 reflections
*c* = 11.5509 (11) Å	θ = 2.9–28.6°
α = 103.081 (2)°	µ = 2.74 mm^−^^1^
β = 96.742 (1)°	*T* = 293 K
γ = 97.860 (1)°	Block, colourless
*V* = 626.43 (11) Å^3^	0.34 × 0.30 × 0.27 mm

### Data collection

**Table:**

Bruker APEXII diffractometer	2315 reflections with *I* > 2σ(*I*)
Radiation source: fine-focus sealed tube	*R*_int_ = 0.035
Graphite monochromator	θ_max_ = 28.6°, θ_min_ = 2.9°
SAINT (Bruker, 2009) scans	*h* = −6→6
4073 measured reflections	*k* = −15→15
3207 independent reflections	*l* = −15→9

### Refinement

**Table:**

Refinement on *F*^2^	Hydrogen site location: inferred from neighbouring sites
Least-squares matrix: full	H-atom parameters constrained
*R*[*F*^2^ > 2σ(*F*^2^)] = 0.055	*w* = 1/[σ^2^(*F*_o_^2^) + (0.1*P*)^2^] where *P* = (*F*_o_^2^ + 2*F*_c_^2^)/3
*wR*(*F*^2^) = 0.166	(Δ/σ)_max_ = 0.027
*S* = 1.11	Δρ_max_ = 0.88 e Å^−^^3^
3207 reflections	Δρ_min_ = −1.39 e Å^−^^3^
164 parameters	Extinction correction: *SHELXL2018/3* (Sheldrick, 2015b), Fc^*^=kFc[1+0.001xFc^2^λ^3^/sin(2θ)]^-1/4^
0 restraints	Extinction coefficient: 0.075 (6)

### Special details

**Table:**

Geometry. All esds (except the esd in the dihedral angle between two l.s. planes) are estimated using the full covariance matrix. The cell esds are taken into account individually in the estimation of esds in distances, angles and torsion angles; correlations between esds in cell parameters are only used when they are defined by crystal symmetry. An approximate (isotropic) treatment of cell esds is used for estimating esds involving l.s. planes.

### Fractional atomic coordinates and isotropic or equivalent isotropic displacement parameters (Å^2^)

**Table:**

	*x*	*y*	*z*	*U*_iso_*/*U*_eq_	
I1	0.12293 (8)	0.97164 (3)	0.27666 (4)	0.0627 (3)	
S1	0.6688 (3)	0.23549 (14)	0.14355 (16)	0.0558 (4)	
C4	0.6072 (11)	0.6675 (5)	0.4017 (4)	0.0400 (10)	
O1	0.7389 (9)	0.6113 (4)	0.0952 (3)	0.0492 (9)	
C6	0.3202 (11)	0.8380 (5)	0.3281 (5)	0.0452 (12)	
N1	0.7570 (11)	0.5853 (5)	0.4409 (4)	0.0502 (11)	
C3	0.5977 (10)	0.6826 (5)	0.2840 (4)	0.0374 (10)	
C2	0.7379 (10)	0.6082 (4)	0.2009 (4)	0.0370 (10)	
C1	0.8777 (12)	0.5224 (5)	0.3644 (5)	0.0473 (12)	
H1	0.976779	0.467243	0.390691	0.057*	
C10	0.8383 (12)	0.3422 (5)	0.0775 (5)	0.0466 (12)	
H10A	0.946662	0.300744	0.022171	0.056*	
H10B	0.705250	0.375036	0.031537	0.056*	
C9	1.0223 (10)	0.4478 (5)	0.1700 (5)	0.0424 (11)	
H9A	1.146034	0.414079	0.219643	0.051*	
H9B	1.130812	0.496966	0.128183	0.051*	
C5	0.4554 (11)	0.7690 (5)	0.2498 (5)	0.0416 (11)	
H5	0.452220	0.780100	0.172336	0.050*	
C7	0.3249 (13)	0.8216 (6)	0.4448 (6)	0.0554 (14)	
H7	0.231139	0.867697	0.498211	0.066*	
O3	0.4727 (11)	0.2855 (6)	0.2052 (7)	0.0900 (19)	
O2	0.8528 (12)	0.1889 (8)	0.2130 (10)	0.139 (4)	
N2	0.8762 (8)	0.5277 (4)	0.2479 (4)	0.0393 (9)	
F1	0.532 (2)	0.1389 (6)	0.0379 (6)	0.167 (4)	
C8	0.4670 (14)	0.7380 (6)	0.4805 (5)	0.0534 (14)	
H8	0.470010	0.728088	0.558397	0.064*	

### Atomic displacement parameters (Å^2^)

**Table:**

	*U*^11^	*U*^22^	*U*^33^	*U*^12^	*U*^13^	*U*^23^
I1	0.0622 (3)	0.0401 (3)	0.0873 (4)	0.01597 (19)	0.0067 (2)	0.0166 (2)
S1	0.0624 (8)	0.0382 (7)	0.0754 (11)	0.0114 (6)	0.0258 (8)	0.0219 (7)
C4	0.049 (2)	0.038 (3)	0.031 (2)	0.001 (2)	0.005 (2)	0.0078 (19)
O1	0.069 (2)	0.049 (2)	0.038 (2)	0.0201 (19)	0.0135 (17)	0.0178 (16)
C6	0.046 (3)	0.033 (3)	0.055 (3)	0.007 (2)	0.006 (2)	0.007 (2)
N1	0.072 (3)	0.048 (3)	0.034 (2)	0.017 (2)	0.003 (2)	0.0161 (19)
C3	0.042 (2)	0.034 (2)	0.036 (2)	0.0027 (19)	0.0040 (19)	0.0112 (19)
C2	0.045 (2)	0.031 (2)	0.036 (3)	0.0049 (19)	0.0046 (19)	0.0147 (19)
C1	0.062 (3)	0.040 (3)	0.039 (3)	0.011 (2)	−0.007 (2)	0.014 (2)
C10	0.064 (3)	0.038 (3)	0.042 (3)	0.010 (2)	0.018 (2)	0.012 (2)
C9	0.044 (2)	0.040 (3)	0.050 (3)	0.014 (2)	0.012 (2)	0.017 (2)
C5	0.050 (3)	0.038 (3)	0.037 (3)	0.006 (2)	0.005 (2)	0.012 (2)
C7	0.067 (4)	0.045 (3)	0.056 (3)	0.012 (3)	0.021 (3)	0.009 (3)
O3	0.078 (3)	0.079 (4)	0.142 (5)	0.028 (3)	0.065 (4)	0.053 (4)
O2	0.073 (4)	0.141 (7)	0.265 (11)	0.040 (4)	0.031 (5)	0.160 (8)
N2	0.044 (2)	0.036 (2)	0.040 (2)	0.0097 (18)	0.0016 (17)	0.0129 (17)
F1	0.280 (10)	0.083 (4)	0.098 (4)	−0.080 (5)	0.054 (5)	−0.009 (3)
C8	0.076 (4)	0.048 (3)	0.041 (3)	0.010 (3)	0.017 (3)	0.015 (2)

### Geometric parameters (Å, º)

**Table:**

I1—C6	2.075 (5)	C2—N2	1.384 (6)
S1—O3	1.390 (6)	C1—N2	1.359 (7)
S1—O2	1.389 (6)	C1—H1	0.9300
S1—F1	1.467 (6)	C10—C9	1.521 (8)
S1—C10	1.748 (5)	C10—H10A	0.9700
C4—C3	1.404 (7)	C10—H10B	0.9700
C4—N1	1.393 (7)	C9—N2	1.462 (6)
C4—C8	1.392 (8)	C9—H9A	0.9700
O1—C2	1.230 (6)	C9—H9B	0.9700
C6—C5	1.363 (8)	C5—H5	0.9300
C6—C7	1.400 (9)	C7—C8	1.367 (9)
N1—C1	1.271 (8)	C7—H7	0.9300
C3—C5	1.387 (7)	C8—H8	0.9300
C3—C2	1.443 (7)		
			
O3—S1—O2	113.9 (5)	C9—C10—H10A	109.1
O3—S1—F1	108.8 (5)	S1—C10—H10A	109.1
O2—S1—F1	110.1 (6)	C9—C10—H10B	109.1
O3—S1—C10	110.6 (3)	S1—C10—H10B	109.1
O2—S1—C10	110.8 (3)	H10A—C10—H10B	107.8
F1—S1—C10	101.9 (3)	N2—C9—C10	114.0 (4)
C3—C4—N1	121.3 (5)	N2—C9—H9A	108.8
C3—C4—C8	118.8 (5)	C10—C9—H9A	108.8
N1—C4—C8	119.8 (5)	N2—C9—H9B	108.8
C5—C6—C7	119.6 (5)	C10—C9—H9B	108.8
C5—C6—I1	120.1 (4)	H9A—C9—H9B	107.7
C7—C6—I1	120.3 (4)	C6—C5—C3	121.0 (5)
C1—N1—C4	116.9 (4)	C6—C5—H5	119.5
C4—C3—C5	119.6 (5)	C3—C5—H5	119.5
C4—C3—C2	119.2 (4)	C8—C7—C6	120.2 (6)
C5—C3—C2	121.2 (4)	C8—C7—H7	119.9
O1—C2—N2	120.0 (5)	C6—C7—H7	119.9
O1—C2—C3	125.0 (4)	C1—N2—C2	121.1 (4)
N2—C2—C3	115.0 (4)	C1—N2—C9	120.1 (4)
N1—C1—N2	126.4 (5)	C2—N2—C9	118.8 (4)
N1—C1—H1	116.8	C7—C8—C4	120.8 (5)
N2—C1—H1	116.8	C7—C8—H8	119.6
C9—C10—S1	112.5 (4)	C4—C8—H8	119.6
			
C3—C4—N1—C1	−2.0 (8)	I1—C6—C5—C3	177.4 (4)
C8—C4—N1—C1	179.1 (6)	C4—C3—C5—C6	−1.2 (8)
N1—C4—C3—C5	−177.4 (5)	C2—C3—C5—C6	179.1 (5)
C8—C4—C3—C5	1.6 (8)	C5—C6—C7—C8	0.8 (9)
N1—C4—C3—C2	2.3 (7)	I1—C6—C7—C8	−176.6 (5)
C8—C4—C3—C2	−178.8 (5)	N1—C1—N2—C2	1.0 (9)
C4—C3—C2—O1	178.3 (5)	N1—C1—N2—C9	−179.1 (6)
C5—C3—C2—O1	−2.1 (8)	O1—C2—N2—C1	−179.9 (5)
C4—C3—C2—N2	−1.0 (7)	C3—C2—N2—C1	−0.6 (7)
C5—C3—C2—N2	178.7 (4)	O1—C2—N2—C9	0.2 (7)
C4—N1—C1—N2	0.3 (9)	C3—C2—N2—C9	179.5 (4)
O3—S1—C10—C9	70.2 (5)	C10—C9—N2—C1	106.4 (6)
O2—S1—C10—C9	−57.1 (7)	C10—C9—N2—C2	−73.7 (6)
F1—S1—C10—C9	−174.2 (6)	C6—C7—C8—C4	−0.4 (10)
S1—C10—C9—N2	−67.0 (5)	C3—C4—C8—C7	−0.7 (9)
C7—C6—C5—C3	0.0 (8)	N1—C4—C8—C7	178.2 (6)

### Hydrogen-bond geometry (Å, º)

**Table:**

*D*—H···*A*	*D*—H	H···*A*	*D*···*A*	*D*—H···*A*
C1—H1···N1^i^	0.93	2.46	3.284 (8)	148
C10—H10*B*···O1^ii^	0.97	2.56	3.493 (7)	160
C10—H10*A*···O1^iii^	0.97	2.45	3.151 (7)	129
C9—H9*A*···O3^iv^	0.97	2.33	3.150 (8)	141
C10—H10*B*···O1	0.97	2.59	3.121 (7)	115

Symmetry codes: (i) −*x*+2, −*y*+1, −*z*+1; (ii) −*x*+1, −*y*+1, −*z*; (iii) −*x*+2, −*y*+1, −*z*; (iv) *x*+1, *y*, *z*.
